# Impact of Plasmodium falciparum and hookworm infections on the frequency of anaemia in pregnant women of rural communities in Enugu, South East Nigeria

**DOI:** 10.11604/pamj.2013.14.27.1925

**Published:** 2013-01-18

**Authors:** Polycarp Uche Agu, Johnbull Sonny Ogboi, Kesiena Akpoigbe, Tochukwu Okeke, Euzebus Ezugwu

**Affiliations:** 1 Department of Obstetrics and Gynaecology, University of Nigeria Teaching Hospital, Ituku-Ozalla, Enugu, Nigeria; 2 University of Camerino, Department of Experimental Medicine and Public Health (Malaria &Human Development), 62032 Camerino (MC), Italy; 3 Society for Family Health, Garki Abuja, Nigeria

**Keywords:** Malaria, hookworm, Enugu, parasites, Nigeria

## Abstract

**Introduction:**

Malaria and hookworm infections are common in sub-Saharan Africa and they increase the prevalence of anaemia in pregnancy with resultant poor pregnancy outcomes. This study was carried out to assess the impact of *Plasmodium falciparum* and hookworm infections on the frequency of anaemia among pregnant women in two rural communities in Enugu, South East Nigeria.

**Methods:**

A cross sectional descriptive study was carried out in a total of 226 women attending antenatal clinics at two rural Primary Health Centres (PHC) from April 2011 to July 2011(each PHC with 113 subjects). Socio-demographic data were collected through a structured questionnaire. Blood and stool samples were evaluated for haemoglobin estimation and malaria parasites, and stool samples examined for parasitic infection in all the women. Data was analyzed using STATA 10 software statistical analysis package. Student t-test was used for comparing mean values and chi square test for comparing categorical variables and level of significance set at p<0.05 and logistic regression was used to identify the risk factors associated with malaria in pregnancy.

**Results:**

The mean age of the women was 27years with range 18 - 38years and SD of 5years. Most of the women were housewives and over 50% in their second trimester. 53% of them had malaria parasites while 27% had hookworm infection. About 40% of the women were anaemic (haemoglobin < 0.001). Similar association was found between hookworm infection and anaemia (p <0.001). Though both malaria and hookworm infections greatly increase the odds for anaemia (AOR 18.06, CI 18.15 -39.99, P<0.001) and (AOR 5.28, CI 2.26-12.38, P<0.001) respectively, the odds for having anaemia in pregnancy was higher for malaria than hookworm infections.

**Conclusion:**

*Plasmodium falciparum* and hookworm infections have significant impact on the high frequency of anaemia in pregnancy in our rural communities. There is need to strengthen the control program that has been in place with an integrated intervention to combat these parasitic infections in our rural communities, with mass distribution of antihelminthics as one of the included relevant methods, among others.

## Introduction

Malaria infection during pregnancy is a major public health problem in tropical and sub-tropical regions throughout the world [1]. This has been most widely evaluated in sub-Sahara Africa where 90% of the global malaria burden occurs and the burden of the infection during pregnancy is caused mainly by *Plasmodium falciparum*, the most common malaria species in Africa [2, 3].

Malaria is a common cause of anaemia in pregnancy in both immune and non-immune individuals [3, 4] with its attendant adverse maternal and peri-natal outcomes such as abortions, low birth weight, still births, and even maternal death [4–6]. In Africa, the prevalence of anaemia in pregnancy has been estimated to be 35 -75% [7] with high malaria rate [8–9]. In Nigeria, malaria is associated with about 11% of maternal death, 25% of infant mortality, and 30% of all childhood deaths [10, 11].

Hookworm infections are also a common cause of iron deficiency anaemia in Nigeria [9, 12] and there is an extensive geographic overlap between *Plasmodium falciparum* and helminth infection in Africa [13]. *P. falciparum* and hookworm co-infection has been shown to have additive impact on anaemia resulting in adverse pregnancy outcomes [14].

There have been few studies on the impact of *P. falciparum* and hookworm co-infection among pregnant women in rural communities where these parasites are endemic. This study was therefore aimed at assessing the impact of *P. falciparum* and hookworm infection on the frequency of anaemia among pregnant women in two rural communities in Enugu State in South East Nigeria.

## Methods

### Study location

The study was carried out in two Primary Health Centers (PHC) located in two different rural communities in Nkanu West Local Government Area (LGA) of Enugu state, Nigeria. These rural communities were randomly selected from the list of rural communities with PHC in the LGA.

The communities were Ogonogoeji Ndiuno Akpugo and Eziokwe Amurri. Both are located over 10 kilometers away from the LGA capital (Agbani) which has a secondary health care facility. These communities share some form of homogeneity in both population and environmental factors. Each having average population of over 2000 inhabitants and surrounded by thick forest with no modern toilet and water drainage facilities which provide conducive environment for breeding mosquitoes.

The people in both communities are mainly peasant farmers and the highest health facility is the PHCs located within the communities. The health centers provide among other primary health care services, antenatal and delivery services to pregnant women within and the surrounding communities.

### Study subjects

This cross sectional descriptive study was conducted among pregnant women at two rural Primary Health Centres (PHC) from April 2011 to July 2011 with each contributing 113 subjects. All the pregnant women coming for antenatal clinic (ANC) on daily basis were enrolled for the study. History, examination and investigations were carried out on all the women.

### Blood sample collection

Safety procedures were adopted in the collection of venous blood samples by swabbing the ante cubital fossae with 70% alcohol and 5mls of blood was drawn into EDTA bottle with sterile hypodermic needle. Thick and thin films were made on clean slides and labeled accordingly as recommended by WHO [7].

### Microscopic examination

The thin films were fixed with methanol and all films were stained with 10% Giemsa stain of pH 7.2 for 30 min as recommended by WHO [14]. The thick films were used to determine the parasite densities while thin films were used to identify the parasite species and infective stages. Stained slides were examined under the light microscope using ×100 objective lens (immersion oil) [14].

### Haemoglobin determination

Five milliliters of blood were collected inside EDTA bottle. Non-heparinized capillary tubes were filled with blood sample from the EDTA bottle. The tip of the capillary tubes were cleaned with cotton wool, sealed and they were arranged inside the haematocrit centrifuge. They were centrifuged for 5 minutes at 12, 000 revolutions per min. The Packed cell volume was determined by using haematocrit reader to read the level of the haemoglobin [7].

### Stool collection and examination

All selected patients were given clean labeled stool containers. The patients were instructed to bring small quantities of their stool specimen the next morning within an hour of passing the stool to the primary health care centre. Wet mounts were prepared from the stool specimen using the direct smear method with normal saline and iodine preparation and the concentration procedure using formol/ether for the identification of helminth eggs as well as protozoan cysts [8].

### Ethical consideration

Approval to conduct the study was obtained from the Primary Health Care centre and the local government authorities of Nkanu West Local Government Area, Enugu State, Nigeria. Verbal consent from the study participants was also obtained before administering the questionnaire.

### Statistical analysis

Data was analyzed using STATA 10 software (Stata Corporation, College Station, Texas) statistical analysis package. A descriptive analysis was done on each variable and bi-variate analysis to assess relationships between the variables. Independent student t-test was used to assess significant mean difference of haemoglobin seen in malaria and hookworm infections, and one-way ANOVA was used to compare the level of anaemia seen in the three trimesters of pregnancy. Chi square (x^2^) was done to compare relationships between categorical variables. Factors that could cause anemia during pregnancy were identified and logistic regression done to factor these covariates. Anaemia in pregnancy was defined as those having haemoglobin concentration of less than 10g/dl, as this is the usual benchmark in most hospitals in Nigeria, and the reference work on it indicated under our discussion.

## Results

**Figure 1:**
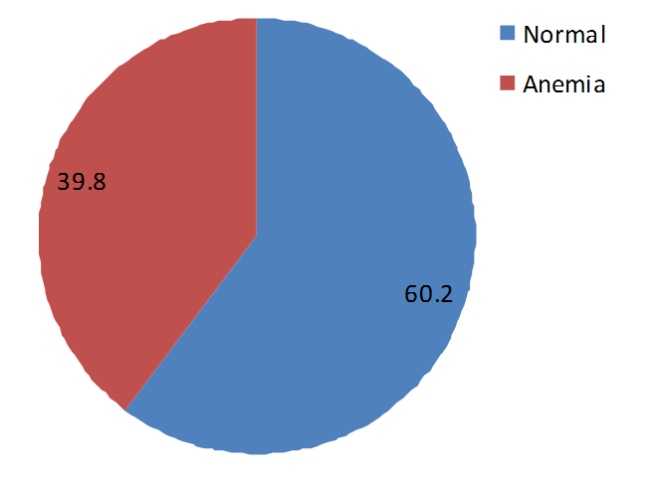
Percentage of anemia in pregnant women

**Figure 2:**
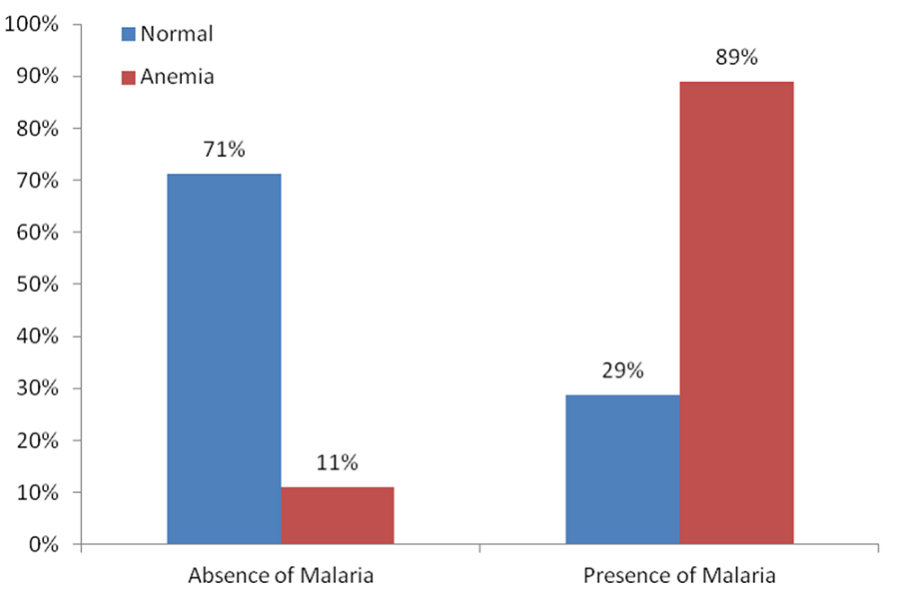
Percentage of anaemia amongst those infected with malaria

**Figure 3:**
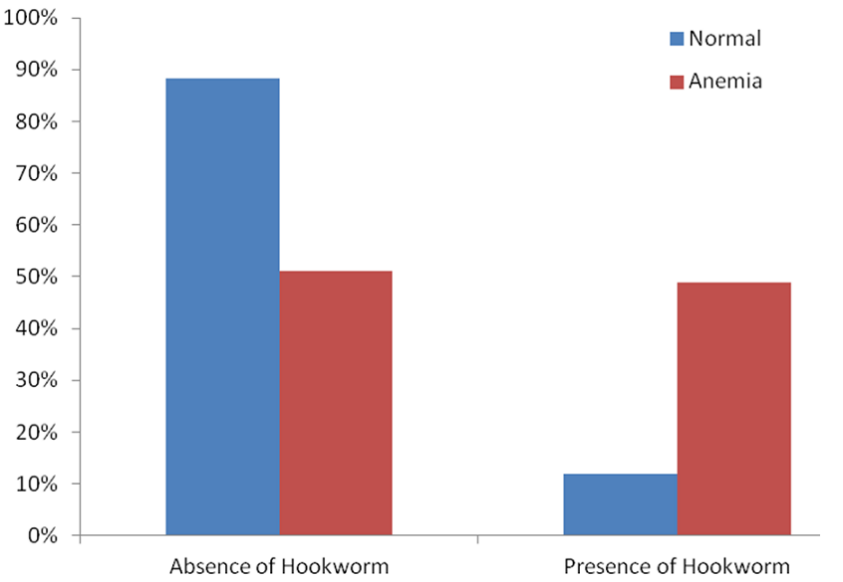
Percentage of anaemia seen amongst those infected with hookworm parasite

The age of individuals in the study ranged from 18-38years with a mean age of about 27years and SD of 5years. Majority of the 226 respondents had no form of education (79%). The highest form of education for most respondents was primary education (15%) (Table 1). Those found in the in the study were mainly housewives in their second trimester (50%). The presence of malaria parasites was observed in the blood of 53% of the individuals that were examined in the study while 27% had hookworm infection, which was the most common infection among those that presented with helminthic infection. The mean haemoglobin concentration is 10.5g/dl (range 8.5g/dl-12g/dl) with about 40% of them being anaemic (haemoglobin less than 10g/dl) (Figure 1). There was a significant difference in the mean hemoglobin concentration (0.84g/dl) between those who have malaria and without malaria (t=9.19, P Table 2 and Figure 2). Similarly, there was an association between hookworm infections and anemia (P Table 2 and Figure 3). Anaemia was found to be more in the second trimester but this relationship was not significant (P=0.406). The major contributors of anaemia in pregnancy were malaria and hookworm infections. The adjusted logistic regression (Table 3) showed that malaria and hookworm infections greatly increases the odds of anemia in pregnancy (AOR 18.06, CI 8.15-39.99, P

**Table 1:** Socio-demographic characteristics of study population

	Frequency	Percentage (%)
**Age (years)**		
0-19*	12	5.3
20-29	150	66.4
30-40	64	28.3
**Education**		
NoSchool	179	79.2
Primary	34	15.1
Secondary	12	5.3
Tertiary	1	0.4
**Occupation**		
Business	5	2.2
Farmer	1	0.4
Housewife	198	87.6
Teacher	1	0.5
Trader	21	9.3
**Marital status**		
Married	222	98.2
Single	4	1.8
**Malaria**		
NoMalaria	107	47.3
Malaria	119	52.7
**Worm infection**		
Ascarislumbricoides	14	6.2
Giardialamblia	1	0.4
Hookworm	60	26.6
Hookworm/Ascaris	2	0.9
Strongyloides	1	0.4
Negative	148	65.5
**Trimester**		
First	65	28.9
Second	113	50.2
Third	47	20.9

**Table 2:** Distribution of Anaemia among demographic characteristics in the study population

Parameters	Normal individuals (Hb>10g/dl) N(%)136(60.2%)	Anaemia individuals (Hb<10d/dl) N(%)90(39.8%)	p-value
**Age**			
0-19	7(5.2)	5(5.6)	0.882
20-29	92(67.7)	58(64.4)	
30-40	37(27.2)	27(30.0)	
**Malaria**			
Absence	97(71.3)	10(11.1)	0.000
Presence	39(28.7)	80(88.9)*	
**Hookworm**			
Absence	120(88.2)	46(51.1)	0.000
Presence	16(11.8)	44(48.9)	
**Trimester**			
First	41(30.3)	24(26.7)	0.406
Second	63(46.7)	50(55.6)	
Third	31(23.0)	16(17.8)	
**Occupation**			
Trader	15(11.0)	6(6.7)	0.619
Housewife	116(85.3)	82(91.1)	
Business	3(2.3)	2(2.2)	
Farmer	1(0.7)	-	
Teacher	1(0.7)	-	
**Education**			
NoSchool	104(76.5)	75(83.4)*	0.498
Primary	22(16.2)	12(13.3)	
Secondary	9(6.6)	3(3.3)	
Tertiary	1(0.7)	-	

* Distribution of anaemia among demographic characteristics in the study population with the relationship between anaemia and malaria parasite being significant with p<0.001.

**Table 3:** Logistic regression analysis for factors associated with anemia in pregnancy

Variables	N(%) 226(100%)	Unadjusted OR(95%CI)	P	Adjusted OR(95%CI)	P
**Age (years)**					
0-19*	12(5.3)				
20-29	150(66.4)	0.88(0.27-2.92)	0.838	0.98(0.26-3.73)	0.977
30-40	64(28.3)	1.02(0.29-3.58)	0.973	1.04(0.23-4.65)	0.958
**Malaria**					
Absence*	107(47.3)				
Presence	119(52.7)	19.90(9.33-42.41)	0.000	18.06(8.15-39.99)	0.000
**Hookworm**					
Absence*	166(73.4)				
Presence	60(26.6)	7.17(3.68-13.98)	0.000	5.28(2.26-12.38)	0.000
**Trimester**					
FirstTrimester*	65(28.9)				
SecondTrimester	113(50.2)	1.36(0.72-2.54)	0.341	1.02(0.43-2.44)	0.961
ThirdTrimester	47(20.9)	0.88(0.40-1.94)	0.754	0.80(0.26-2.43)	0.689
**Occupation**++					
Trader*	21(9.29)				
Housewife	198(87.6)	1.77(0.66-4.76)	1.13	1.35(0.37-4.92)	0.648
Business	5(2.2)	1.67(0.22-12.67)	0.49	4.94(0.85-28.70)	0.075
**Education**					
NoSchool*	179(79.2)				
Primary	34(15.0)	0.76(0.35-1.63)	0.474	0.75(0.27-2.04)	0.571
Secondary	12(5.3)	0.46(0.12-1.77)	0.260	0.47(0.11-2.08)	0.321

* Base Case;

++ Farmers, Teachers in occupational category and Tertiary in educational category were dropped because of insufficient observations for running a logistic regression.

## Discussion

Improving the understanding of the effect of malaria and hookworm infections ( and possibly co-infections also) on the frequency of anaemia in pregnancy may help in the design of appropriate management strategies targeted at reducing adverse pregnancy outcomes often associated with these infections. The high prevalence of 53% for *P.falciparum* parasitaemia found in our study is close to 60% reported by Fuseni et al in Ghana [8]. This is higher than 42.6% reported by Ndyomugyenyi et al in Uganda [15], but lower than 81% reported by Ekejindu et al in Anambra, Nigeria [9]. The high *Plasmodium* parasitaemia in the communities are not surprising as the communities under study are surrounded with thick forest and have poor water drainage facilities which provide conducive environment for breeding mosquitoes. This situation is compounded by low use of insecticide treated nets among the rural dwellers. The prevalence of 27% for hookworm infection found in this study is similar to 30% reported by Ozumba et al in Enugu, Nigeria [12], but higher than 23% reported by Ndyomugyenyi in Masindi, Uganda [15], 17% by Ekejindu in Anambra, Nigeria [9], and 20.7% by Fuseni et al in Ghana [8]. The practice of defeacating in the farm land and bushes and the low socioeconomic status in the communities could be the reasons for the high prevalence of intestinal helminthiasis observed in the study.

The 40% prevalence of anaemia in this study is consistent with 40.4% reported by Dim and Onah in Enugu [5], but higher than 28% by Ndyomugyenyi etal in Masindi Uganda [15]. The anaemia prevalence would have been much higher if World Health Organization criterion (haemoglobin < 11g/dl) was used in this study. In practice, most hospitals in Nigeria use a lower level of haemoglobin [16], which showed that serious harm to mother and fetus did not occur until the haemoglobin value was below 10g/dl. The high prevalence of anaemia in our study is in tandem with the high prevalence of *P.falciparum* and hookworm infections. The significant relationship between *Plasmodium* parasite and hookworm infections on one side and anaemia on the other side is obvious in our findings in this study that 89% and over 50% of 153 pregnant women with malaria and hookworm infections respectively were anaemic. The frequency of anaemia is further aggravated by the additive effect of *P.falciparum* and hookworm co-infections in this study. This is similar to findings in the previous studies [8, 9, 17]. The pathogenesis of anaemia by *P.falciparum* is attribu to haemolysis of the infected red blood cells, decreased red blood cell production and other potential causes such as rupture of infected red blood cells with pathological effects of accompanying events, removal of uninfected red cells due to antibody sensitization, marrow hypoplasia seen in acute infections of malaria which may cause decreased production of red cells and consequent anaemia, among few other causes. Hookworm infections results in iron loss from the infested intestinal lumen. It has been estimated that a load of 1000 hookworm ova per gram of faeces is associated with loss of 1mg of iron per day [18]

Poor nutritional intake (though not evaluated in this study) common among the rural dwellers may also have contributed to the high prevalence of anaemia among these pregnant women. Interestingly, but as reported in other studies from Nigeria [5, 19–21], most of the anaemia found in this study were of mild variety. Though mild to moderate anaemia are generally tolerated, it may adversely affect the sense of wellbeing of these rural women who are involved in farming and other physically exhaustive work resulting in fatigue with consequent decrease in work capacity. Our study did not find any significant contribution of age, education and occupation to anaemia frequency among the rural pregnant women.

## Conclusion

We conclude that the prevalence of anaemia in pregnancy is high in our rural communities. *Plasmodium falciparum* and hookworm infections have significant impact in the high prevalence. We suggest that an integrated interventional program to combat both malaria and helminthes infections in our communities should be strengthened to have significant health impact. Routine screening and treatment of both malaria and hookworm infections should be emphasized in our antenatal care. This will help prevent anaemia and reduce maternal and perinatal morbidity and mortalities in our communities.
